# Prediction of outcome in patients with suspected acute ischaemic stroke with CT perfusion and CT angiography: the Dutch acute stroke trial (DUST) study protocol

**DOI:** 10.1186/1471-2377-14-37

**Published:** 2014-02-25

**Authors:** Tom van Seeters, Geert Jan Biessels, Irene C van der Schaaf, Jan Willem Dankbaar, Alexander D Horsch, Merel JA Luitse, Joris M Niesten, Willem PTM Mali, L Jaap Kappelle, Yolanda van der Graaf, Birgitta K Velthuis

**Affiliations:** 1 Department of Radiology, University Medical Center Utrecht, Heidelberglaan 100, HP E01.132, 3584 CX, Utrecht, The Netherlands; 2 Department of Neurology, Utrecht Stroke Center, Brain Center Rudolf Magnus, University Medical Center Utrecht, Utrecht, The Netherlands; 3 Department of Radiology, Rijnstate Hospital, Arnhem, The Netherlands; 4 Julius Center for Health Sciences and Primary Care, University Medical Center Utrecht, Utrecht, The Netherlands

**Keywords:** Stroke, Ischaemia, Infarct, Prediction, CT perfusion, CT angiography

## Abstract

**Background:**

Prediction of clinical outcome in the acute stage of ischaemic stroke can be difficult when based on patient characteristics, clinical findings and on non-contrast CT. CT perfusion and CT angiography may provide additional prognostic information and guide treatment in the early stage. We present the study protocol of the Dutch acute Stroke Trial (DUST). The DUST aims to assess the prognostic value of CT perfusion and CT angiography in predicting stroke outcome, in addition to patient characteristics and non-contrast CT. For this purpose, individualised prediction models for clinical outcome after stroke based on the best predictors from patient characteristics and CT imaging will be developed and validated.

**Methods/design:**

The DUST is a prospective multi-centre cohort study in 1500 patients with suspected acute ischaemic stroke. All patients undergo non-contrast CT, CT perfusion and CT angiography within 9 hours after onset of the neurological deficits, and, if possible, follow-up imaging after 3 days. The primary outcome is a dichotomised score on the modified Rankin Scale, assessed at 90 days. A score of 0–2 represents good outcome, and a score of 3–6 represents poor outcome. Three logistic regression models will be developed, including patient characteristics and non-contrast CT (model A), with addition of CT angiography (model B), and CT perfusion parameters (model C). Model derivation will be performed in 60% of the study population, and model validation in the remaining 40% of the patients. Additional prognostic value of the models will be determined with the area under the curve (AUC) from the receiver operating characteristic (ROC) curve, calibration plots, assessment of goodness-of-fit, and likelihood ratio tests.

**Discussion:**

This study will provide insight in the added prognostic value of CTP and CTA parameters in outcome prediction of acute stroke patients. The prediction models that will be developed in this study may help guide future treatment decisions in the acute stage of ischaemic stroke.

## Background

Stroke is the fourth leading cause of death and the most common cause of long term disability [1]. Around 20% of stroke patients die within one year after the stroke event [1]. Prediction of long-term clinical outcome in acute ischaemic stroke is important, but not easy when based on patient characteristics, clinical findings and non-contrast CT (NCCT). Improving outcome prediction may help determine the optimal treatment strategy in the acute stage, when clinicians need to weigh possible benefit and harmful side effects of treatment. For treatment with intravenous thrombolysis with recombinant tissue type plasminogen activator (IV-rtPA) for example, time since stroke onset is the main safety criterion for patient selection. Additional imaging techniques might improve risk stratification.

CT imaging plays a critical role in the acute stage of stroke assessment as it is faster and more feasible than MRI and available 24/7 in stroke clinics worldwide. The NCCT can easily be extended with CT perfusion (CTP) and CT angiography (CTA) within a 5- to 10-minute examination [2] and has been successfully incorporated into acute stroke imaging protocols [3-5]. CTP and CTA may offer important parameters for predicting stroke outcome. CTP offers the possibility to differentiate reversible from irreversible ischaemic brain tissue and can give information on the permeability of the blood brain barrier [2,6-11]. On CTA, the location and extent of the arterial occlusion, collateral circulation, extracranial stenosis, and ischaemic changes within brain tissue can be visualised [12-17]. These parameters, including patient characteristics and NCCT findings, have been described separately in studies with MRI and/or CT, but have not been combined in one study to assess their combined predictive value.

The Dutch acute Stroke Trial (DUST) aims to improve prediction of outcome in patients with acute ischaemic stroke. For this purpose, we will assess the additional predictive value of CTP and CTA to the conventional determinants of patient characteristics and NCCT findings. We will develop an individualised prediction model for clinical outcome after stroke based on patient characteristics and CT parameters. This prediction model may help guide clinicians in making better treatment decisions.

## Methods/design

### Study design

**Figure 1:**
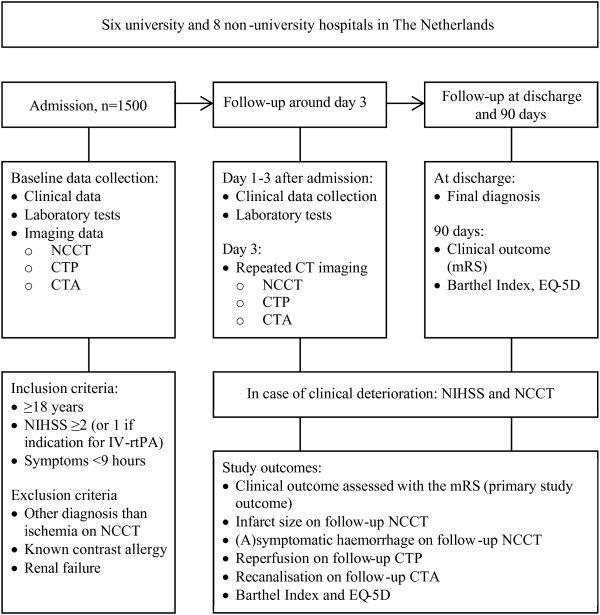
Study procedures and outcomes.

The DUST is a prospective multicentre observational cohort study with the aim to include 1500 patients with a suspected acute ischaemic stroke. Six university and 8 non-university hospitals in The Netherlands participate in this study. On admission, baseline characteristics are collected, laboratory testing is performed, and patients undergo CT imaging consisting of NCCT, CTP and CTA (see Figure 1). The following 3 days, additional patient characteristics and results of laboratory tests are collected. When possible, follow-up imaging is performed around 3 days after admission to evaluate if there is an infarct and if other admission imaging findings have changed. Information on the final diagnosis (or death) is collected at discharge. Clinical outcome, as primary study outcome, is assessed by the modified Rankin Scale (mRS) at 90 days.

### Inclusion and exclusion criteria

All patients with a clinical diagnosis of acute ischaemic stroke (both anterior and posterior circulation) are included if the following criteria are met: 1) older than 18 years, 2) National Institutes of Health Stroke Scale (NIHSS) ≥2, or 1 if an indication for IV-rtPA is present (e.g. isolated aphasia), 3) acute neurological deficit of less than nine hours of duration. Patients with an unknown time of symptom onset, i.e. patients who wake up or are found with stroke symptoms, can be included if the time from going to sleep or being last seen without symptoms, until imaging is less than 9 hours. Patients are excluded from the study if another diagnosis on admission NCCT explains the neurological deficits (e.g. intracerebral haemorrhage, subarachnoid haemorrhage, or tumour). Furthermore, patients cannot be included if they have a known contrast allergy or previously known renal failure at the time of admission.

### Baseline clinical and laboratory data collection

#### Clinical data collection at baseline

After inclusion, details on stroke symptoms are collected, including time of symptom onset, time of arrival at the hospital, and stroke severity determined by the NIHSS [18]. As patients with an already poor functional status prior to the stroke symptoms are less likely to have a good outcome, pre-existing functional status is summarised in an mRS score [19]. Patient characteristics including blood pressure, pulse rate, length, weight, temperature, any medication use, and medical history are also collected. Regarding medical history, information is recorded about the presence of cardiovascular risk factors such as hypertension, diabetes mellitus, hyperlipidemia, prior vascular disease (including stroke and myocardial infarction), alcohol intake, smoking, and family history of vascular disease in first grade relatives. Furthermore, data on whether or not the patient is treated with IV-rtPA, intra-arterial thrombolysis (IAT), or mechanical thrombectomy, and time from stroke onset to treatment is collected.

#### Laboratory data collection at baseline

Laboratory tests performed at baseline include serum creatinine, glucose, hemoglobin, thrombocytes, C-reactive protein, and INR.

### Baseline CT acquisition and post-processing

#### Acquisition

On admission to the hospital, all patients undergo NCCT, CTP and CTA. All imaging is performed on 40- to 320-detector CT scanners (Philips, Siemens, GE, Toshiba). NCCT parameters are 120 kV, 300–375 mAs, and a 5 mm reconstructed slice thickness.

CTP scans are generally performed with 80 kV and 150 mAs, and are also reconstructed as 5 mm contiguous axial slices. Forty ml of non-ionic contrast material is injected intravenously with a flow of 6 ml/s followed by 40 ml of saline with a flow of 6 ml/s. Images are acquired every 2 seconds for 50 seconds after initiation of contrast injection, followed by 1 image every 30 seconds from 60 to 210 seconds. CTP coverage ranges from 40 mm to full brain coverage and covers at least the basal ganglia up to the lateral ventricles to ensure that both Alberta Stroke Program Early CT Score (ASPECTS) levels are included [20-22]. CTP coverage can be lowered to cover the cerebellum to the occipital lobes if a stroke in the posterior circulation is expected, covering all three levels of the posterior circulation ASPECTS (pcASPECTS) [17].

For the CTA, from aortic arch to cranium vertex, 50–70 ml of non-ionic contrast material is injected intravenously with a flow of 6 ml/s followed by 40 ml of saline with a flow of 6 ml/s. The CTA scan delay after intravenous contrast injection is calculated for each patient individually from time to peak arterial enhancement on CTP, or by a trigger based on Hounsfield unit threshold measurement of contrast enhancement in the aortic arch.

#### Post-processing

The scan data from all participating hospitals are collected centrally in the University Medical Center Utrecht. Post-processing of the CTP and CTA scans is performed using commercially available software on an Extended Brilliance Workstation (version 4.5, Philips Healthcare).

The software for CT perfusion post-processing has been validated previously, and applies Gaussian curve fitting by least mean squares to obtain mathematical descriptions of the time-enhancement curves [4,23-25]. After calculation of the cerebral blood volume (CBV) from the area under the time attenuation curve, the mean transit time (MTT) is computed by using a deconvolution operation [26]. Subsequently, cerebral blood flow (CBF) is calculated according to the central volume principle (CBF = CBV/MTT). Based upon previously reported MTT and CBV thresholds, threshold-defined penumbra and infarct core maps are calculated as well as the penumbra/infarct core index [4]. The total ischaemic area is defined as a relative measure of MTT ≥145% compared to the contralateral (unaffected) hemisphere. Within this ischaemic area, the infarct core is separated from the penumbra by an absolute threshold value of CBV <2.0 ml/100 g.

For the CTA, thin slice data is reviewed, with interactive maximum intensity projection (MIP) and multiplanar reconstruction (MPR) possibilities, to assess the anatomy and pathology of the extracranial and intracranial vessels. Additional automated vessel segmentation is performed for the carotid and vertebral arteries to assess the degree of stenosis.

### Baseline CT imaging data collection

All admission scans are assessed by an observer with at least five years of experience in neurovascular imaging (from a pool of three observers). Except for the side of symptoms, observers are blinded for all clinical information, including follow-up scans and clinical outcome.

For NCCT, presence of a hyperdense vessel sign is determined and early ischaemic changes are assessed with ASPECTS or pcASPECTS [12,20-22]. An area is considered ischaemic on NCCT if there is parenchymal hypoattenuation with or without swelling of the brain [21,22]. Areas with isolated cortical swelling, but without hypoattenuation, are not considered ischaemic [22]. Presence of haemorrhage, atrophy, white matter lesions, or old infarct is also noted. On CTA source images (CTA-SI), areas with diminished contrast enhancement are considered ischaemic and are assessed with (pc)ASPECTS [12]. Next, the CTA is evaluated for location and extent of arterial occlusion, leptomeningeal collateral circulation, circle of Willis configuration, presence of dissection or sinus thrombosis, as well as presence and degree of carotid or vertebrobasilar stenosis, and presence of intracranial atherosclerosis. Measurement of carotid or vertebrobasilar stenosis is performed according to the NASCET method [27]. For CTP, presence of a perfusion deficit is visually assessed on CBV, CBF, MTT, time to peak (TTP), and penumbra/infarct core maps, and evaluated with (pc)ASPECTS [11,28]. Reliability of ASPECTS has been shown to be fair for NCCT, moderate for CTA-SI and excellent for CTP [29], and inter-observer agreement is excellent for assessment of site of occlusion, good for collateral circulation, and good to excellent for carotid stenosis [15,30].

### Follow-up clinical and laboratory data collection

#### Follow-up clinical data collection

Temperature data is collected on day 1 to 3 after admission, and blood pressure and pulse rate on day 3. In case of clinical deterioration, severity of the worsening symptoms is determined by a new NIHSS assessment (plus an additional NCCT). At discharge, information on the final diagnosis, flow territory, and cause of ischaemia (such as large vessel disease, cardiac origin, dissection, etc.) is collected. Clinical outcome is assessed with the mRS at 90 days [31]. At 90 days, the Barthel Index and EQ-5D are also collected [32,33].

#### Follow-up laboratory data collection

Laboratory tests performed on day 1 include (fasting) glucose, HbA1c, HDL- and LDL-cholesterol, and triglycerides. On day 3, data on serum creatinine, glucose (non-fasting), and C-reactive protein are collected.

### Follow-up imaging data collection

When possible, patients undergo follow-up CT imaging 3 days after admission, or earlier if patients are discharged. Standard follow-up imaging consists of NCCT, CTP and CTA with an imaging protocol identical to the baseline imaging protocol. Radiological evidence of an infarct is determined on the follow-up NCCT. If an infarct is present, final infarct volume is calculated and (pc)ASPECTS is allocated. Any visible haemorrhage is classified according to the ECASS classification [34]. Reperfusion is evaluated on the CTP and recanalisation on the CTA. NCCT suffices if there is any reason not to administer intravenous contrast material. MRI can also serve as follow-up imaging if clinically indicated (preferably with diffusion-weighted, T2-FLAIR and T2-weighted images, and MR angiography). In case of clinical deterioration, a NCCT is performed to detect intracranial haemorrhage (plus an additional NIHSS).

### Study outcome measures

The primary outcome measure of this study is poor clinical outcome assessed by the mRS at 90 days [31]. Collection of the 90 day mRS is performed by a trained research nurse or neurologist by telephone interview [35]. A score of 0–2 represents good outcome, and a score of 3–6 represents poor outcome [36]. Secondary outcome measures include infarct size, symptomatic and/or asymptomatic haemorrhage on follow-up NCCT, reperfusion on follow-up CTP, recanalisation on follow-up CTA, and Barthel Index and EQ-5D at 90 days.

### Data entry and monitoring

Individual clinical and laboratory patient data is collected by the local investigators and supporting staff in the participating hospitals, and entered in a password-protected electronic online case report form. Participating centres can only access patient data from patients included in their own specific hospital. Prior to access to the online case report form, all investigators are trained in its use. To minimise missing or wrong (implausible) data, investigators in the coordinating centre check the database and missing values are retrieved where possible.

### Analysis

#### Candidate predictors

Prediction models will be developed to investigate the primary study outcome. In these models, we only include variables with an expected strong predictive value for the outcome based on prior literature. Candidate predictors that will be included in the prediction models are divided into 3 groups: (1) patient characteristics and NCCT, (2) CTA, and (3) CTP predictors.

1) Patient characteristics include age [37-39], stroke severity (NIHSS) [38-40], time from symptom onset to imaging, mRS prior to current stroke symptoms [19], admission glucose level [41-43], and treatment (yes/no for IV-rtPA/IAT/mechanical thrombectomy) [44,45]. NCCT predictors are early ischaemic changes (ASPECTS or pcASPECTS) [17,20,21], and presence of a hyperdense vessel sign [19,46].

2) CTA predictors include ischaemic changes on CTA-SI (ASPECTS or pcASPECTS) [12,13,17], location and extent of arterial occlusion [16], leptomeningeal collaterals [15,47], and presence of severe ipsilateral carotid or vertebrobasilar artery stenosis (>70%) or occlusion [48].

3) CTP predictors include perfusion deficits on CBV and MTT maps (ASPECTS or pcASPECTS) [11], and penumbra/infarct core index [4].

#### Model derivation

For the development of the prediction rule, three logistic regression models will be developed in the derivation cohort consisting of 60% of the study population. Selection of patients for the derivation cohort based on date of inclusion. The first model will include patient characteristics and NCCT parameters (Model A), reflecting the situation in which CTP and CTA are not performed as part of standard stroke workup. The second model will consist of Model A + CTA predictors (Model B). The third model will include Model B + CTP predictors (Model C). Model selection and shrinkage of the model coefficients will be performed [49]. The optimal penalty factor will be determined with bootstrapping. Single imputation will be used to decrease missing values. In addition to true missing values, biologically improbable values are also considered missing values. To minimise the effect of outliers, continuous predictors will be truncated at the 1^st^ and 99^th^ percentile [49].

#### Model validation

Model performance will be validated in the remaining 40% of the study population. Discrimination of the models, i.e. the ability of the models to discriminate between patients with good and patients with poor outcome, will be assessed with receiver operator characteristics (ROC) analysis and corresponding area under the curve (AUC) [50]. Calibration of the models, i.e. agreement between predicted outcomes and observed outcomes, will be evaluated with calibration plots. In these plots, patients are divided into quantiles according to their predicted risk. For each quantile, the mean predicted probability is compared to the mean observed outcome. Goodness-of-fit will be tested with Hosmer-Lemeshow tests. Likelihood ratio tests will be used to compare the difference in the models for statistical significance. Superiority of the models will be determined by using a combination of the results of the ROC analyses, calibration plots and Hosmer-Lemeshow tests, and likelihood ratio tests. A nomogram or score chart will be developed for the best model to facilitate use by clinicians.

### Sample size

Based on previously published data on acute stroke in The Netherlands, we expect approximately half of the 1500 patients to have a poor clinical outcome [51]. Hence, in the derivation cohort consisting of 900 patients, we anticipate a poor outcome in about 450 patients. In total, we plan to include 15 variables in the most extensive model (model C). We are able to reliably estimate the weight of the predictors, as we have more than the (arbitrary) amount of 10 events per predictor, allowing for enough power for unexpected findings [52]. As we expect about half of the patients to undergo some form of treatment (IV-rtPA/IAT/mechanical thrombectomy) we still have enough power to assess outcome in both the treatment and non-treatment group [53]. The large study size also ensures sufficient numbers for subgroup analyses, such as stroke subtype, posterior circulation stroke, and stroke complications.

### Ethical considerations

The medical ethics committee of the University Medical Center Utrecht approved the DUST. The study is conducted according to the principles of the Declaration of Helsinki and in accordance with the Dutch Medical Research Involving Human Subjects Act. Patients are only included in the DUST after written informed consent is obtained from the patient or his/her legal representative. Clinical data collection, laboratory testing, and CT imaging at admission is performed as part of the routine clinical work-up of stroke patients. Informed consent is obtained before any further study related tests or procedures are performed. Because stroke patients may have a compromised ability to consent, the capacity to consent is established first. In patients with a compromised ability to consent, written informed consent is obtained from their legal representative. Patients who die before informed consent can be obtained are an exception. Because it is undesirable to burden the relatives with this request, the medical ethics committee waived the need for informed consent in these patients.

## Discussion

Prediction of clinical outcome in patients with symptoms of acute ischaemic stroke is not easy when based on patient characteristics and NCCT alone. To the best of our knowledge, the combined prognostic value of CTP and CTA parameters in addition to clinical and NCCT parameters has never been investigated in one single study.

In our study design we aim to resemble clinical practice as much as possible for the sake of clinical applicability and generalizability. Our study domain consists of patients with a suspected acute ischaemic stroke at the time of admission to the hospital, and therefore patients with an unknown time of symptom onset, such as wake-up strokes, and patients who turn out to have a stroke mimic, are also included. As treatment decisions are made in the acute stage when time is critical, prediction of outcome as guideline for further treatment is needed for all patients who are suspected of acute ischaemic stroke, and not just for selected patients with middle cerebral artery stroke who are eligible for treatment. The large study population enables analysis of sufficient candidate predictors, and also permits further division, such as between anterior and posterior circulation strokes and whether treatment is given or not. The candidate predictors selected for our prediction models can be determined quickly in the clinical setting, enabling their use in the acute stage. For this multicentre study CT imaging is performed on scanners from different vendors. While this may result in more variation in the imaging results, it improves generalizability to other hospitals, which is of major importance for prediction studies. Imaging assessment is performed by a single experienced observer, which reflects the clinical practice of most hospitals. Lastly, while many studies use surrogate endpoints such as infarct volume or recanalization, using clinical outcome at 90 days remains the most relevant outcome for stroke patients.

In conclusion, this study will provide insight in the added prognostic value of CTP and CTA parameters in outcome prediction of acute stroke patients. The prediction models that are developed in this study may guide future treatment decisions in the acute stage.

## Abbreviations

ASPECTS: Alberta stroke program early CT score; AUC: Area under the curve; CBF: Cerebral blood flow; CBV: Cerebral blood volume; CTA: CT angiography; CTA-SI: CTA source images; CTP: CT perfusion; DUST: Dutch acute stroke trial; IAT: Intra-arterial thrombolysis; IV-rtPA: Intravenous recombinant tissue type plasminogen activator; MIP: Maximum intensity projection; mRS: Modified Rankin scale; MTT: Mean transit time; MPR: Multiplanar reconstruction; NCCT: Non-contrast computed tomography; NIHSS: National institutes of health stroke scale; pcASPECTS: Posterior circulation ASPECTS; ROC: Receiver operator characteristics; TTP: Time to peak.

## Competing interests

The authors declare that they have no competing interests.

## Authors’ contributions

BV, LJK, WM, and YvdG designed the study with BV as the principal investigator. AH, BV, GJB, IvdS, JN, JWD, ML and TvS are responsible for data collection and analysis. All authors have read and approved the final manuscript.

## Pre-publication history

The pre-publication history for this paper can be accessed here:

http://www.biomedcentral.com/1471-2377/14/37/prepub
